# The Bioartificial Heart: Our Mission to Mars

**DOI:** 10.21470/1678-9741-2025-0044

**Published:** 2025-03-17

**Authors:** Gabriel R. Liguori

**Affiliations:** 1 TissueLabs SA, Bellinzona, Switzerland

Few endeavors capture the imagination quite like the ambition of sending humans to Mars. It is a challenge so vast, so complex, and so groundbreaking that it pushes the boundaries of what we believe humanity can achieve. Yet, within the field of regenerative medicine, there exists an equally audacious vision: the creation of a bioartificial heart for transplantation. Like the journey to Mars, this goal is a convergence of science, engineering, and persistence. It requires not only ambition but also a strategy to make the impossible possible.

While we may not have rockets in our labs, there is much to be learned from Elon Musk’s approach to SpaceX. His journey offers a compelling framework for how to tackle seemingly insurmountable challenges. If we hope to one day create bioartificial hearts, we must think not just like scientists but also like builders, engineers, and entrepreneurs.

SpaceX’s early years were defined by pragmatism wrapped in bold vision. Musk knew that the ultimate goal - Mars colonization - was far beyond the company’s initial capabilities. Instead, he focused on building the foundations. The Falcon 1 rocket, while far from Mars-ready, proved that a small team could design, build, and launch a rocket. This first step secured contracts, revenue, and credibility, which in turn funded SpaceX’s bigger ambitions^[1-3]^.

Similarly, in our field, the path to a bioartificial heart begins not with the heart itself but with the tools and technologies that enable progress. For the last five years, we have focused on creating 3D bioprinting systems and advanced biomaterials that empower researchers to print simple tissues in the lab. These building blocks are already unlocking new possibilities for regenerative medicine in hundreds of institutions across the globe and laying the groundwork for what comes next.

This approach is not just pragmatic - it is essential. Before tackling the complexity of a fully functional heart, we must first refine our ability to create smaller, yet impactful tissues.

After the success of Falcon 1, SpaceX moved quickly to scale up. Falcon 9 wasn’t just a rocket, it was a reusable workhorse that made regular launches economically viable. But crucially, Falcon 9 also enabled another major breakthrough: Starlink, the satellite network that would revolutionize global connectivity while providing sustainable revenue for the company^[2,3]^.

In our journey, this stage is reflected in our focus on creating cardiac tissues for pharmaceutical development. Just as Starlink leveraged SpaceX’s rocket technology to generate resources while advancing a broader vision, our engineered cardiac tissues will provide pharmaceutical companies with human-relevant models for drug testing. These tissues transform how drugs are tested and developed, offering more accurate, human-relevant models for research - all while pushing us closer to our ultimate goal.

With Falcon 9 firmly established, SpaceX turned its attention to the next frontier: human spaceflight. In 2020, the company’s Crew Dragon spacecraft became the first commercial vehicle to carry astronauts to the International Space Station, marking a historic moment for private space exploration^[3]^. This step wasn’t just about reaching space - it was about demonstrating SpaceX’s ability to support life in extreme environments.

In regenerative medicine, the equivalent step is clinical application. As our bioprinting technology matures, we will move into the creation of simple but vital cardiovascular tissues, such as blood vessels and heart valves. These are the first bioprinted tissues that will be implanted in human patients, demonstrating their viability and setting the stage for more complex applications. Just as SpaceX proved it could send astronauts into orbit, this step will prove that bioprinted tissues can function in real-world clinical settings.

And then comes the ultimate goal - the one that started it all. For SpaceX, that goal is landing humans on Mars. For us, it is the creation of a fully functional bioartificial heart, capable of being transplanted into a human patient. Like space colonization, this endeavor requires solving countless technical and biological challenges: creating complex myocardial tissue, integrating vascularization at scale, and ensuring post-transplantation long-term function. These are challenges no one has solved yet, but with each stage of progress, we are getting closer. The bioartificial heart represents not just an advancement in regenerative medicine, but a fundamental shift in how we approach organ transplantation and the treatment of heart disease. It is our Mars landing - the achievement that will change the future of healthcare forever.

What made SpaceX’s journey remarkable was its ability to turn skeptics into believers. In the early days, reusable rockets were dismissed as unrealistic. Today, they are the industry standard. Similarly, the idea of a bioartificial heart may feel like science fiction now, but history shows us that persistence and a methodical approach can make the unimaginable a reality.

SpaceX’s success stems largely from Musk’s visionary insistence on the company’s ability to control its own destiny. By building everything in-house, from rocket engines to spacecraft, SpaceX freed itself from the limitations of traditional aerospace contractors. This vertical integration enabled speed, cost control, and innovation^[1-3]^.

We believe the same principle applies to regenerative medicine. Achieving the bioartificial heart will require expertise in every aspect of its development - from bioprinting technologies to cell and tissue engineering, all the way to clinical applications. This problem cannot be solved piecemeal; it requires a unified effort driven by a singular vision.

When SpaceX launched its first rockets, the odds were stacked against it. But it wasn’t just the rockets that made people believe - it was the clarity of the mission and the conviction of those who dared to take part in it. Today, SpaceX’s story is told as a triumph of persistence and ingenuity, a reminder that even the loftiest dreams can be realized with the right strategy.

In regenerative medicine, we stand at a similar crossroad. The creation of a bioartificial heart is one of the most ambitious medical challenges ever conceived, and it will not happen overnight. But with each step forward - each technological advance and clinical milestone - we will turn what once seemed impossible into a tangible reality.

To those who share our belief in this vision: the journey is under way. The tools are being built, the foundations are being laid, and the path forward is clearer than ever. The question is not whether this can be achieved, but how long it will take - and who will dare to be part of it.

Mars is no longer out of reach. Neither is the bioartificial heart.
